# Clinical spectrum of cleidocranial dysplasia: a case report

**DOI:** 10.1186/1757-1626-1-377

**Published:** 2008-12-08

**Authors:** Rajeev Kumar Garg, Prachi Agrawal

**Affiliations:** 1 Dental Surgery, 35 Kishanpole, Jaipur 302001, Rajasthan, India; 2 Intern, Govt. Dental College & Hospital, Jaipur 302016, Rajasthan, India

## Abstract

**Background:**

Cleidocranial dysplasia is a developmental anomaly of the skeleton and the teeth. This condition may be inherited, be transmitted as dominant characteristics in either sex, or even may appear spontaneously. It presents with skeletal defects of several bones, like partial or complete absence of clavicles, late closure of the fontanels, presence of open skull sutures and multiple wormian bones.

**Case presentation:**

In this case report, we describe an otherwise healthy 30 year-old male with a chief complaint of missing anterior maxillary and Mandibular teeth.

**Conclusion:**

Cleidocranial dysplasia is very rare in occurrence, incidence being 1: 1,000,000. Since early diagnosis of cleidocranial dysplasia is essential for initiating the appropriate treatment approach, clinicians should be aware of the characteristic features. We report a case of cleidocranial dysplasia because of its rarity.

## Background

Cleidocranial dysplasia is a rare congenital defect of autosomal dominant inheritance, [1,2] primarily affecting bones that undergo intra-membranous ossification, i.e. generally the calvarian but also the clavicular bones. It is also known as Marie and Sainton disease, Mutational dysostosis and Cleidocranial dysostosis [3]. Cleidocranial dysplasia was first described by Pierre Marie and Paul Sainton in 1898, [4] since then, over 1000 cases have been documented in the medical literature [5].

Cleidocranial dysplasia presents with skeletal defects of several bones, the most striking of which are partial or complete absence of clavicles, late closure of the fontanels, presence of open skull sutures and multiple wormian bones [1,6]. Late closure of fontanels is also a feature of Basal cell nevus syndrome and Crouzon syndrome, but together with other characteristic features, cleidocranial dysplasia can be easily differentially diagnosed. The skull base is dysplastic and reduced in growth resulting in increased skull width leading to brachycephaly and hypertelorism [5]. Delayed closure of anterior fontanel and metopic sutures result in frontal bossing.

Thoracic cage is small and bell shaped with short ribs. Typically, clavicles are underdeveloped to varying degrees and in approximately 10 percent of cases, are completely absent. This allows excessive mobility of the shoulder girdle. Other bones may also be affected including long bones, the vertebral column, the pelvis and the bones of hands and feet [7].

Characteristically, patients with cleidocranial dysplasia, show prolonged retention of deciduous dentition and delayed eruption of permanent teeth. Adults with cleidocranial dysplasia have mixed dentition in their oral cavities. In addition, patients with this condition, frequently show a large number of unerupted supernumerary teeth, often mimicking a premolar. As many as 63 unerupted supernumerary teeth have been documented in one patient [8]. Maxilla is also underdeveloped along with ill-formed paranasal sinuses. This condition is of clinical significance to every dentist due to the involvement of the facial bones, altered eruption patterns and multiple supernumerary teeth.

## Case report

A 30 year-old male presented to Dental Surgery clinic, Jaipur, with a chief complaint of missing anterior teeth in upper and lower jaws. He told that he hadn't had any teeth in these regions of the jaws since childhood after his milk teeth shed away. Due to these missing teeth, he had an unpleasant smile that resulted in a psychological trauma to him while communicating with society members. He insisted for rehabilitation of his missing teeth and smile.

**Figure 1:**
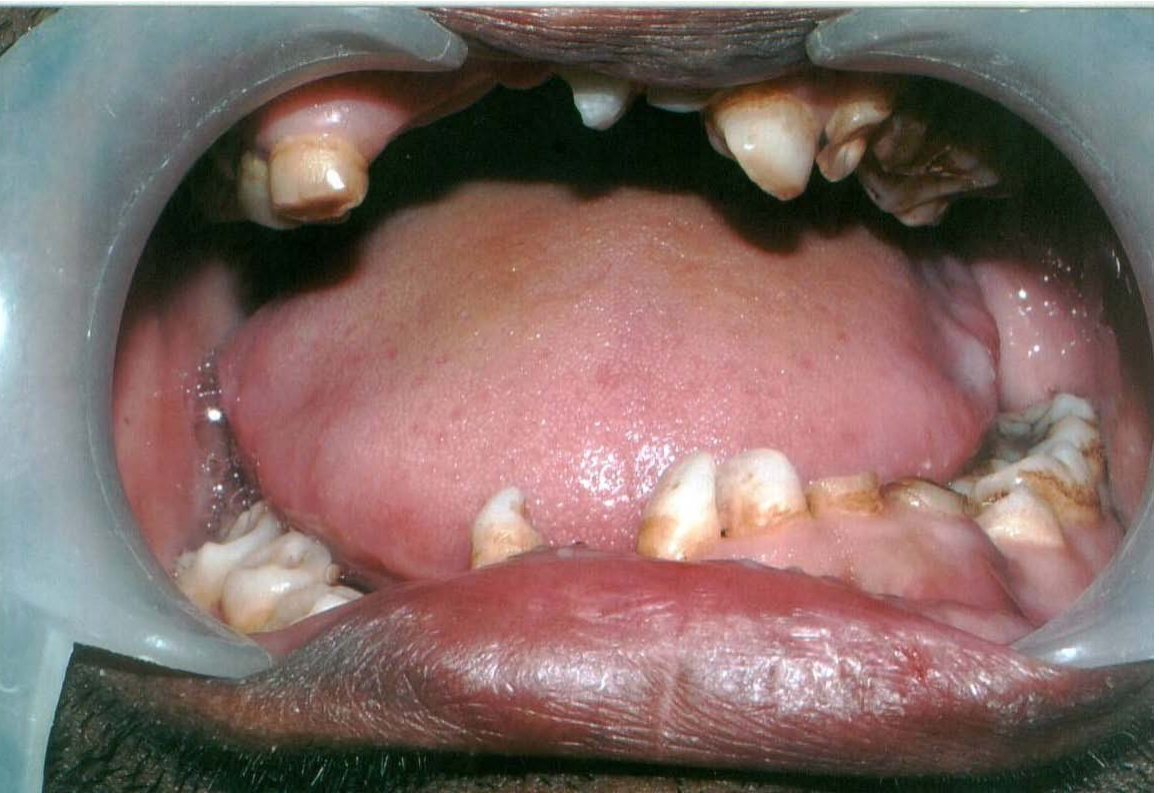
Oral view showing numerous over-retained deciduous and some missing teeth.

Examination of the oral cavity revealed multiple over-retained deciduous teeth and some missing teeth, particularly in anterior maxilla and mandible on right side (Figure 1). But, not much thought was given to these findings at the initial examination and it was decided that panoramic and bitewing radiographs were required to evaluate the patient's overall dentition.

**Figure 2:**
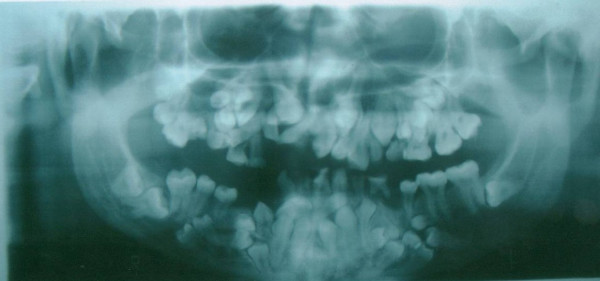
Panoramic view of the jaws showing multiple unerupted supernumerary teeth mimicking premolar, missing gonial angles and underdeveloped maxillary sinuses.

On evaluating the panoramic radiograph, the classical signs of cleidocranial dysplasia were immediately recognized (Figure 2). The patient had approximately 64 teeth in his both of the jaws. Some of the teeth were erupted but most of them were unerupted and mimicking a premolar in shape. Gonial angles on both sides of mandible were missing and maxillary sinuses were underdeveloped.

**Figure 3:**
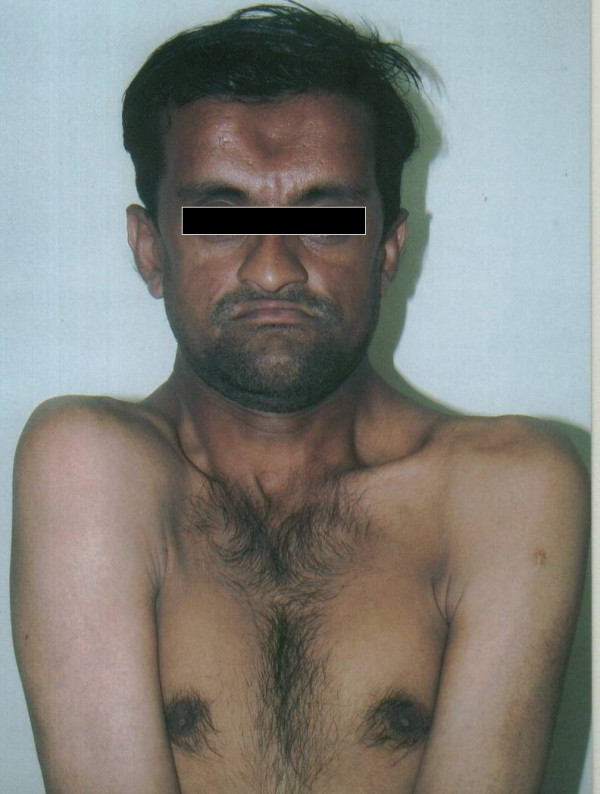
Facial profile view of the patient demonstrating hypermobility of the shoulder girdles and frontal bosselation.

The patient was then, asked to attempt to place his shoulders adjacent to each other to check for the incomplete clavicle bone formation and this attempt demonstrated more than normal mobility of the shoulder girdle (Figure 3). Upon re-examination of the face, it was found that he also had the classical symptoms of frontal bosselation, hypertelorism and mid face deficiency.

**Figure 4:**
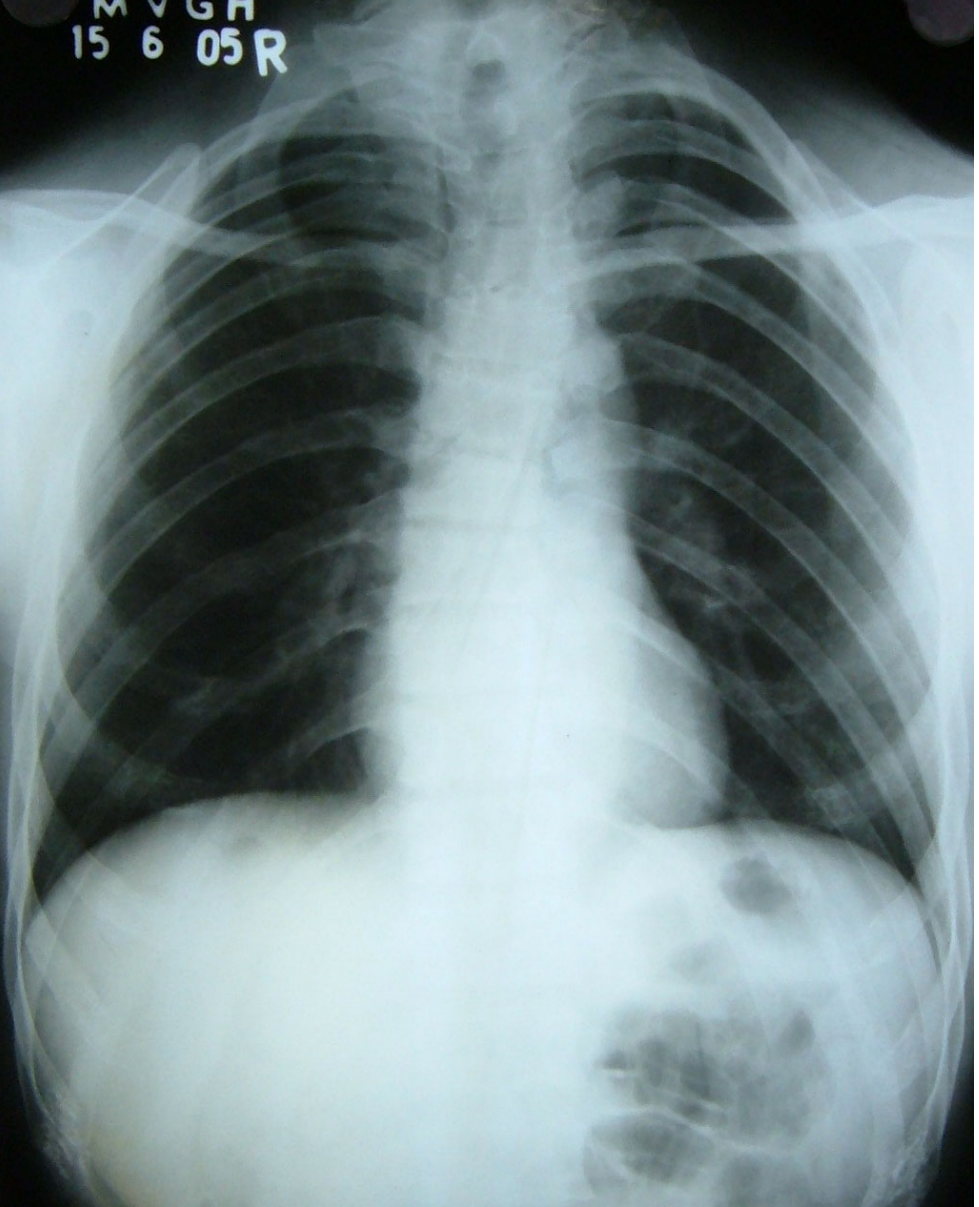
Chest radiograph (PA View) of the patient showing thinning and hypoplasia of the clavicles and bell shaped rib-cage.

**Figure 5:**
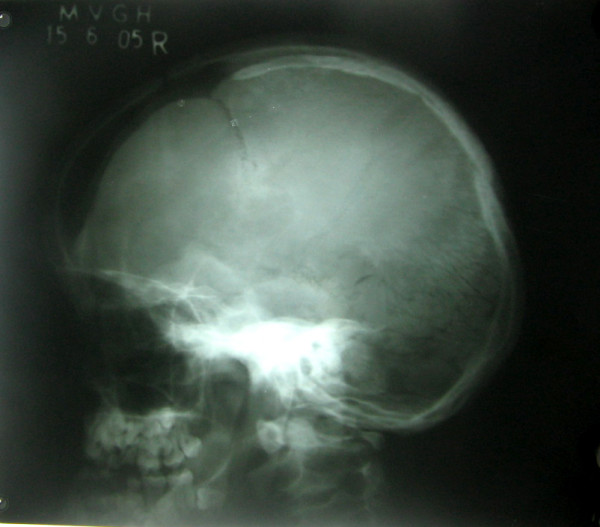
Lateral skull radiograph showing open skull sutures, large fontanels, multiple wormian bones and underdeveloped paranasal sinuses.

Chest radiograph (PA View) confirmed the clavicular hypoplasia and bell shaped rib-cage (Figure 4). Besides this, skull radiograph (Lateral view) demonstrated open skull sutures, delayed closure of fontanels and multiple wormian bones (Figure 5). It also showed poorly formed paranasal sinuses and zygomatic complex.

A diagnosis of cleidocranial dysplasia was confirmed and the patient was referred to prosthodontic department of Govt. Dental College & Hospital, Jaipur for prosthetic rehabilitation where he was given dentures for his missing teeth.

## Discussion

One of the most presumptive clinical findings of cleidocranial dysplasia is hypermobility of the shoulders [3]. Due to partial or complete absence of the clavicles, the shoulders can be brought forward to close proximity to the chest. Bosselation is present owing to the failed closure of the metopic sutures and anterior fontanel [1]. Hypertelorism [9] is a common finding and maxilla and paranasal sinuses present as being underdeveloped [10].

Dental findings are characterized by decreased eruptive force of both primary and permanent dentitions, prolonged retention of primary teeth [2] and increase in odontogenesis leading to excessive number of supernumerary teeth [11]. There is a predisposition to develop numerous supernumerary teeth, particularly in mandibular premolar and maxillary anterior regions [12]. Other than delayed, the permanent molars generally erupt without incident [1,3]. Removal of primary or supernumerary teeth does not usually promote eruption of unerupted permanent teeth [3].

The radiographic evaluation of patients is the most important and reliable means to confirm the diagnosis, since radiological findings of cleidocranial dysplasia are pathognomonic, i.e. broad sutures, large fontanels persisting into adulthood, numerous wormian bones and numerous unerupted supernumerary teeth[1,3].

Cervical or thoracic vertebral defects, supernumerary ribs, thoracic and lumber scoliosis, kyphosis or lordoisis, pelvic bony abnormalities and anomalies of phalangeal, tarsal, metatarsal, carpal and metacarpal bones are all systemic findings [3].

The suggested treatment for dental complications of cleidocranial dysplasia is:

1. Fabrication of dentures over the unerupted teeth, and

2. Removal of teeth as they erupt, for very little bone structure would be left if supernumerary, impacted and unerupted teeth were all extracted at once.

## Conclusion

The clinical findings of cleidocranial dysplasia, although present at birth, are often either missed or diagnosed at a much later time. Some cases are diagnosed through incidental findings by physicians, treating patients for unrelated conditions. Cleidocranial dysplasia may be identified by family history, excessive mobility of shoulders and radiographic pathognomonic findings of the chest, skull and jaws.

## Competing interests

The authors declare that they have no competing interests.

## Authors' contributions

RKG and PA, both analyzed and interpreted the patient data, made the diagnosis and both gave a major contribution in writing the manuscript. All authors read and approved the final manuscript.

## Consent

This is to be declared that a written informed consent was obtained from the patient for publication of this case report and accompanying images. A scanned copy of the consent is attached here.
